# Femoral Neck Non-union Treated using Compression Screw with or without Gluteus Medius Trochanteric Flap: A Case Series of Ten patients

**DOI:** 10.5704/MOJ.2111.023

**Published:** 2021-11

**Authors:** WI Faisham, I Munajat, AA Salim

**Affiliations:** Department of Orthopaedics, Universiti Sains Malaysia, Kubang Kerian, Malaysia

**Keywords:** femoral neck non-union, femoral neck fracture, trochanteric flap, myo-osseous flap, sliding compression screw

## Abstract

Non-union is a challenging complication following a femoral neck fracture. Inability to achieve anatomical reduction and compression over the fracture leads to non-union. We reported a 10-case series of femoral neck non-union treated with sliding compression screw and anti-rotational screw with or without gluteus medius local trochanteric flap. When compression could not be achieved and a gap was present over the non-union site, a gluteus medius trochanteric flap was used to enhance the union. Surgeries were performed as a single-stage procedure through the Watson Jones approach. The initial implants were removed, followed by fracture reduction, during which the varus deformity was corrected, and the neck length was preserved as much as possible. Patients were advised for strict non-weight bearing until the presence of trabecular bone crossing the fracture on the radiographs. Union was achieved at three months in all cases. Patients undergoing surgery without trochanteric flap had normal abduction strength, and the neck length was maintained. All cases had no significant loss of function. Patients with trochanteric myo-osseous flap had neck shortening with weak abductors with MRC grade 4. Two out of 10 cases developed avascular necrosis of the femoral head before intervention. One case progressed to collapse of the femoral head requiring implant removal. This and the femoral neck shortening, caused this patient to have weak abductors and a positive Trendelenburg gait. We observed that delayed surgery leads to neck shortening and fracture gap requiring trochanteric myo-osseous flap to achieve union.

## Introduction

A femoral neck fracture is associated with avascular necrosis (AVN), implant failure, and non-union of the fracture ranging between 10-30%, leading to a re-operation rates as high as 20%^1^. A successful union depends significantly on the anatomical reduction and the compression over the fracture. The femoral neck non-union is still common and challenging after a femoral neck fracture. Head preserving procedures should be considered in young patients for the benefit of long-term functional outcome.

We reported the outcomes of 10 cases of femoral neck non-union treated with sliding compression screw with or without gluteus medius local trochanteric flap.

## Case Report

Ten cases of the established non-union neck of the femur were treated with a sliding compression screw in our centre. In cases where primary compression could not be achieved and the fracture gap was still present, a gluteus medius trochanteric flap and autologous trochanteric bone graft were used to augment the non-union site.

There were seven males and three females, aged ranging from 15 to 37 years old with an average age of 22. All patients with an established diagnosis of femoral neck non-union secondary to failure of the previous implant were included in this case series. The initial treatments were cannulated screws in six, reconstruction nail in one and proximal femur locking compression plate (LCP) in one case. Three patients had the neck of femur fracture following interlocking nail surgery, in which two of them were managed by cannulated screw fixation using miss the nail technique to the neck, which subsequently failed. In contrast, the other one was addressed by replacing the interlocking nail with a sliding compression screw for the fractured neck and plate for the fractured shaft. Two non-union cases had pre-operative radiological evidence of femoral head AVN, which were managed with DHS stabilisation and trochanteric myo-osseous flap.

**Fig. 1:**
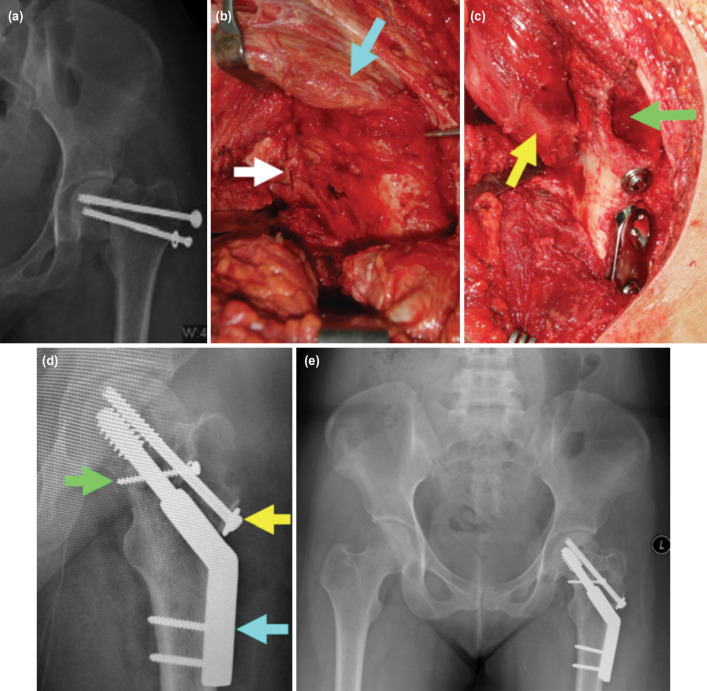
(a) Photos showing the initial screw fixation of case no. 2 with non-union of the femoral neck, varus deformity and neck shortening, (b) open reduction thru Watson Jones approach, anterior one-third of the gluteus medius (blue arrow), reduction of the neck fracture (white arrow), correction of the varus deformity and the neck length, (c) myo-osseous trochanteric flap transferred to the non-union site (yellow arrow), additional bone graft needed was harvested from the trochanter underneath the osteotomised osseous flap (green arrow), (d) post-operative radiograph showing satisfactory reduction and correction maintained with sliding compression screw (blue arrow) and anti-rotational screw (yellow arrow), one cancellous screw (green arrow) secured the myo-osseous flap at the non-union site, (e) radiograph seven years post-surgery with union of the femoral neck without evidence of AVN.

All surgical procedures were performed as a single-stage procedure through the Watson Jones approach. After initial implants (Fig. 1a) were removed, the fracture was opposed to correct the varus deformity (Fig. 1b). Fracture reduction was achieved by manual traction and a sharp-pointed clamp placed over the superior fragment of the neck and over the lateral trochanter to achieve an adequate neck-shaft angle. Two threaded wires were used as primary stabilisation at the femoral neck's inferior and superior parts to minimise the helicopter effect while reaming the neck for sliding compression screw. A 6.5mm cannulated screw which acted as an anti-rotational implant, was subsequently inserted over the superior part of the neck parallel to the sliding compression screw (Fig. 1d and e).

The anterior one-third of the gluteus medius muscle was split proximally as far as 4cm and distally to its insertion on the greater trochanter's anterior portion, which was later osteotomised (Fig. 1b). The proximally based myo-osseous flap, which consisted of the gluteus medius and its distal bony attachment, was mobilised to the superior anterior part of the femoral neck at the fracture region (Fig. 1c). We created a cortical window at the neck into which the myo-osseous flap would be placed and stabilised with a cancellous screw. Any gap over the non-union site was filled with the cancellous bone graft harvested from the trochanteric area underneath the osseous flap previously osteotomised (Fig. 1c). The flap was free of tension through all range of hip motion. The viability of the flap was assessed from free bleeding of its muscle and bone.

Patients were advised for strict non-weight bearing until trabecular bone crossing of the fracture site was noted on the radiograph. Neck shaft angle and evidence of implant loosening were assessed as an indirect sign of union. In cases of avascular necrosis prior to intervention, patients were advised to continue partial weight-bearing with a single crutch for six months to prevent femoral head collapse.

**Fig. 2:**
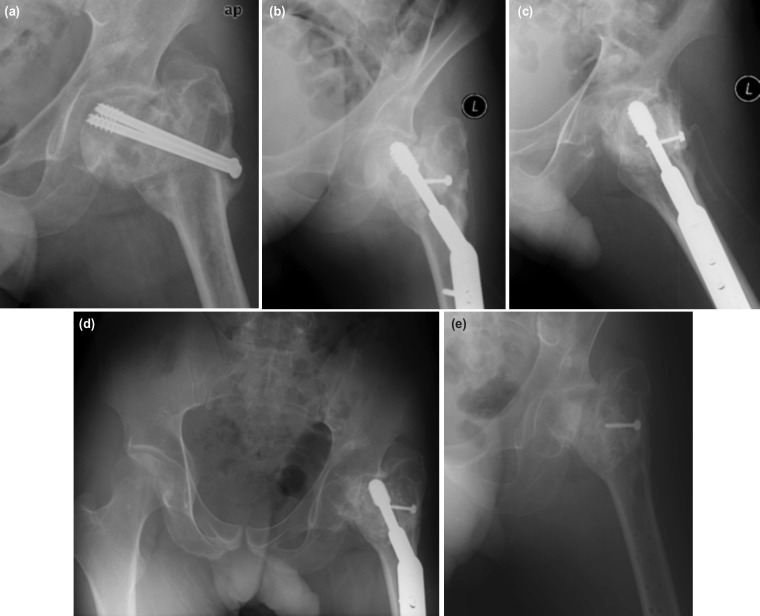
(a) Photos showing the case no. 5 with non-union of the femoral neck, loosening of the screws, resorption of neck and AVN of the femoral head evidenced by sclerosis of the entire head, (b, c) after removal of the previous implants, dynamic hip screw and anti-rotational screw were inserted, and myo-osseous trochanteric flap was performed leading to successful union, (d) however the AVN progressed with collapse of the femoral head five years later, (e) warranting an early implant removal. The patient was ambulating with Trendelenburg gait and required single crutch in daily activity. He was planned for an early joint replacement later.

Union of the femoral neck was achieved at three months in all cases. In cases undergoing DHS with an anti-rotational screw without gluteus medius flap, the abduction strength was almost comparable to the contralateral normal side, and the neck length was maintained. All cases remained functional with no significant loss of function. All patients with trochanteric flap had active hip abduction of MRC grade 4. One case (case no. 5 as in Table I) who had preoperative avascular necrosis with the short femoral neck as shown in Fig. 2a, b, c, was complicated with head collapse as in Fig. 2d. This patient had weak abductor and positive Trendelenburg test due to femoral neck shortening and femoral head collapse. Case no. 5 was considered a failure, which was one out of 10 cases highlighted in this series since the femoral head had total collapse due to initial AVN warranting implant removal as in Fig. 2e and required early joint replacement.

**Table I:** Data of 10 patients with neck of femur non-union

Case	Age/ Sex/Side	Primary stabilisation method	AVN occurrence	Trochanteric myo-osseous flap used	Duration for final surgery with DHS and anti-rotational screw (months)	Follow-up (years)	Outcomes
1	37/F/R	ILN for femoral shaft fracture but missed NOF fracture. Removal of ILN and replaced with DHS for fractured neck and DCP for fractured shaft	No	No	5 months	10 years	United, No AVN LLD 1 cm, Abductor MRC 5
2	25/F/L	2 cannulated screws	No	Yes	7 months	7 years	United, No AVN LLD 1cm, Abductor MRC 4
3	22/M/L	Reconstruction nail for ipsilateral NOF and shaft fractures	Yes	Yes	24 months	4 years	United, AVN head LLD 2 cm, Abductor MRC 3
4	20/M/R	3 cannulated screws for NOF fracture and DCP for ipsilateral shaft fracture	No	Yes	1 month	12 years	United, No AVN LLD 1 cm, Abductor MRC 4
5	30/M/L	2 cannulated screws	Yes	Yes	13 months	5 years	United with short neck. AVN head with collapse. Implant removed. Trendelenburg gait.
6	20/M/R	3 cannulated screws. Supracondylar plate for distal femur fracture	No	Yes	4 months	6 years	United, No AVN No LLD, Abductor MRC 4
7	20/M/R	Missed NOF fracture after ILN femoral shaft. 2 cannulated screw fixation thru missed nail technique	No	No	2 months	4 years	United, No AVN No LLD
8	30/M/L	2 cannulated screws. Retrograde nail for ipsilateral shaft fracture	No	No	3 months	5 years	United, No AVN LLD 1 cm
9	37/F/L	NOF fracture was not recognized after ILN shaft fracture.	No	No	3 months	3 years	United, No AVN LLD 1 cm
10	32/M/L	Proximal femur LCP for NOF and per trochanteric fractures	No	No	3 months	3 years	United, No AVN LLD 1 cm

Abbreviations – AVN: avascular necrosis, NOF: neck of femur, ILN: interlocking nail, DHS: dynamic hip screw, DCP: dynamic compression plate, LLD: limb length discrepancy, MRC: Medical Research Council Scale for Muscle Strength, LCP: locking compression plate

**Fig. 3:**
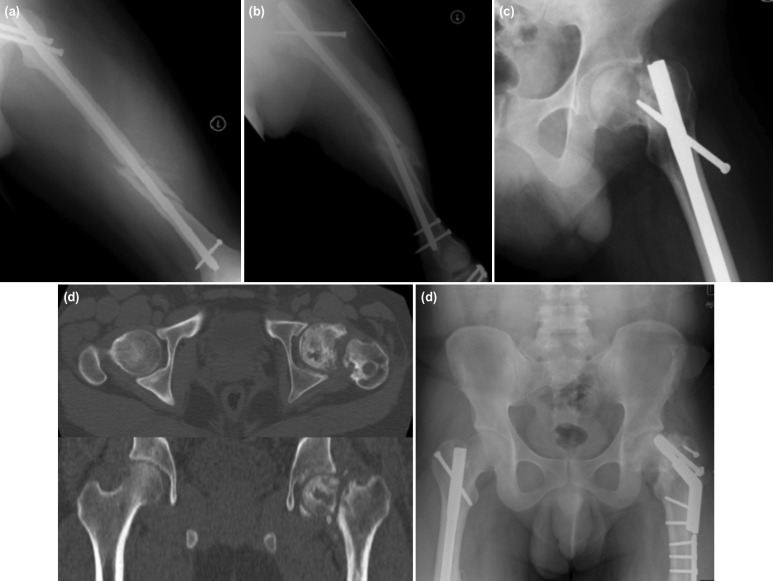
(a) Photos showing the initial reconstruction nail used to address the shaft and the femoral neck fractures in case no.3, (b) second traumatic event caused implant failure to the previously fixed femoral neck fracture, (c) radiological evidence of non-union over the femoral neck, (d) imaging from the CT scan showed more obvious radiological evidences of non-union and AVN of the femoral head, (e) radiograph four years after sliding compression screw and myo-osseous trochanteric flap showed that the neck already united, however there was head AVN and shortening of the femoral neck. Patient had 2cm shortening and MRC grade 3 hip abductor.

Another one case (case no. 3) who had a failure of reconstruction nail as shown in Fig. 3a, b, c, and femoral head AVN as in Fig. 3d showed evidence of revascularisation even though the final surgery was performed slightly later, which was two years after the first surgery. The patient was also ambulating pain-free after four years of final surgery despite a 2cm shortening. The radiograph four years after the surgery showed that the neck fracture had united. However, there was AVN of the femoral head and shortening of the femoral neck, as shown in Fig. 3e. We observed that delayed surgery could lead to neck shortening and fracture gap that required trochanteric myo-osseous flap to achieve union. Most patients treated with trochanteric myo-osseous flap had shortening with weak abductor of grade 4.

## Discussion

The technique described is a simple surgery that can be performed by a general orthopaedic surgeon for treatment of neck of femur non-union to minimise the need for early hip replacement in a young patients. In cases where the fracture gap is still present, augmentation with trochanteric muscle pedicle graft is an option to improve vascularity and fusion mass over the non-union area. We observe that the neck length is still maintained in cases where the surgery is performed relatively early due to minimal bone resorption. Thus, a simple DHS implant can compress the fracture without the need for a trochanteric flap.

The treatment of femoral neck non-union in a young patient is still a head preserving procedure. Stabilisation with sliding hip screw in femoral neck non-union is recommended in the absence of malalignment, shortening and before deformity has occurred. Augmentation with an anti-rotational screw is necessary to prevent rotation. Temporary transfixation with wires across the fracture should be done before reaming for the sliding hip screw to prevent rotation. Reaming of the head and neck may provide internal autogenous grafting and augment healing over the non-union site. Wu *et al* report satisfactory outcome using a sliding compression screw without subtrochanteric valgus osteotomy and recommend it as a standard^1^. Early open correction of varus deformity and primary compression, as shown in previous studies and this series, provides a good outcome as neck length was maintained^1,2^.

Neck shortening in non-union reflects the severity of femoral neck resorption, and it is due to delayed diagnosis and intervention. Correction of neck shortening and neck-shaft angle will maintain the abductor moment arm and hip function. Open approach to achieve the above correction requires the clearing of the fibrous tissue, which creates the gap and then bridge the non-union with the graft^3^. Free vascularised fibula graft (FVFG) has shown to be an effective method to solve non-union of the femoral neck in young patients, even in the presence of avascular necrosis. Good to excellent mid-term hip score function is reflected by the solid union and anatomical hip moment arm^3^. This procedure requires specialised microsurgical expertise and is technically demanding, and is not reproducible in many hospitals in developing countries. Rotational muscle pedicle graft from trochanter or ischium is another option to induce solid union over the fracture and some degree of revascularisation.

Das De and Balasubramaniam described the anterior part of the trochanter could be mobilised with gluteus medius without damaging its vascular supply^4^. Anatomical dissection found that ascending branch of the superior gluteal artery skirts the iliac crest's rim to supply the gluteus medius and minimus muscles 4cm above the trochanter^4^. The trochanteric pedicle can be shifted without tension to the superior anterior neck and provides vascularity for head avascular necrosis^5^. The gluteus medius trochanteric flap has the advantage of being less invasive, less demanding surgery, and not involving vascular anastomosis. Harvesting a cancellous bone graft from underneath the osteotomised area of the trochanter is also hassle-free.

In our series, all five cases augmented with myo-osseous trochanteric flap had a bone gap at their non-union site. Therefore, we believed the myo-osseous trochanteric flap added value to achieving union by accentuating the vascular supply over the bone gap filled with cancellous bone graft. However, this procedure may not provide sufficient vascularity to the femoral head with pre-existing AVN.
